# The complete mitochondrial genome of the longfin dragonfish *Tactostoma macropus* (Stomiiformes: Stomiidae)

**DOI:** 10.1080/23802359.2018.1464411

**Published:** 2018-04-23

**Authors:** Minoru Ijichi, Tsuyoshi Takano, Masumi Hasegawa, Haruka Yashiki, Kazuhiro Kogure, Shigeaki Kojima, Susumu Yoshizawa

**Affiliations:** Atmosphere and Ocean Research Institute, The University of Tokyo, Kashiwa, Chiba, Japan

**Keywords:** Deep-sea, gene rearrangement, mitogenome, pelagic fish

## Abstract

The complete mitochondrial genome (mitogenome) was determined for the longfin dragonfish *Tactostoma macropus*, which is the first for the genus and the third within the family Stomiidae. The mitogenome sequence is 17,690 bp in length containing 2 ribosomal RNA genes, 22 transfer RNA genes, 13 protein-coding genes, and a control region, as in most fishes. The gene order of *T. macropus* showed an unreported deviation from the typical vertebrate one. Phylogenetic reconstruction using the maximum likelihood method placed *T. macropus* in the monophyletic Stomiiformes. Three stomiid species were recovered as a moderately supported clade in the phylogenetic tree.

The barbeled dragonfishes of the family Stomiidae (Stomiiformes) are representative deep-sea pelagic predators (Kenaley 2012). Stomiid taxonomy has attracted considerable attention due to the rarity of these fishes. In addition, the remarkable phenotypical and ecological specialization of these species to life in dark waters includes the use of not only blue but also far-red bioluminescence to communicate (e.g. Kenaley et al. 2014). Phylogenetic analysis based on mitochondrial genome (mitogenome) data would improve our understanding of the evolutionary history of stomiids. However, stomiid mitogenomes are available for only 2 – *Chauliodus sloani* and *Stomias atriventer* – of the more than 280 species (Miya et al. 2001; Aguilar et al. 2018). In the current study, we determined the complete mitochondrial DNA sequence of the longfin dragonfish *Tactostoma macropus*; this is the first complete mitogenome for the genus and the fifth within the order Stomiiformes.

A beam-trawl haul yielded a single *T. macropus* at station KANO4 of the R/V *Shinsei-maru* cruise KS-17-6 (off Otsuchi, Iwate, Japan: 39°24.97′–39°25.00′N, 143°14.48′–143°19.58′E; depth, 2,161–2,414 m). The specimen was stored at −30 °C until dissection, and then fixed with 99% ethyl alcohol; vouchered DNA (171127_DNA1) was deposited at the Atmosphere and Ocean Research Institute, The University of Tokyo. Total genomic DNA was extracted from the photophore under the right eye, and paired-end sequencing (2 × 300 bp) was performed in an Illumina MiSeq sequencer. Bases with a quality score lower than 20 were removed from sequence reads, and then trimmed reads shorter than 127 bp were discarded using Sickle (Joshi and Fass 2011). Remaining reads were assembled using SPAdes version 3.10.1 (Bankevich et al. 2012). The assembled mitogenome sequence was annotated using the MitoFish website (Iwasaki et al. 2013); some annotations were corrected manually.

The complete mitogenome sequence of *T. macropus* (DDBJ/EMBL/GenBank accession no. LC377784) is 17,690 bp in length containing 2 ribosomal RNA (12S and 16S) genes, 22 transfer RNA genes (tRNAs), 13 protein-coding genes, and a control region (CR, D-loop). *Tactostoma macropus* shows a deviation from the typical vertebrate gene order in the Cyt *b*-tRNA^Thr^-tRNA^Pro^-CR region: gene rearrangement resulted in its specific order of tRNA^Pro^-CR-Cyt *b*-tRNA^Thr^ (see Satoh et al. 2016). In addition, noncoding sequences are present between tRNA^Glu^ and tRNA^Pro^ (217 bp) and between tRNA^Thr^ and tRNA^Phe^ (151 bp). All protein-coding genes but COI (GTG) and ATP6 (ATT) use ATG as the start codon; TAA, TAG, TA–, T––, AGG, and AGA are found as stop codons. The ATT start codon of ATP6 does not occur in 250 previously determined fish mitogenomes (Satoh et al. 2016). The overall base composition is 25.1% for A, 24.5% for T, 31.4% for G, and 19.0% for C.

Partitioned maximum likelihood analysis placed *T. macropus* in the monophyletic Stomiiformes (bootstrap percentage [BS] = 100%; Figure 1). The family Stomiidae was recovered as a moderately supported clade (BS = 63%), although a recent mitogenomic phylogeny has suggested its non-monophyly (Aguilar et al. 2018). Further investigation with dense taxonomic sampling will shed new light on the phylogenetic status of the family.

**Figure 1.**
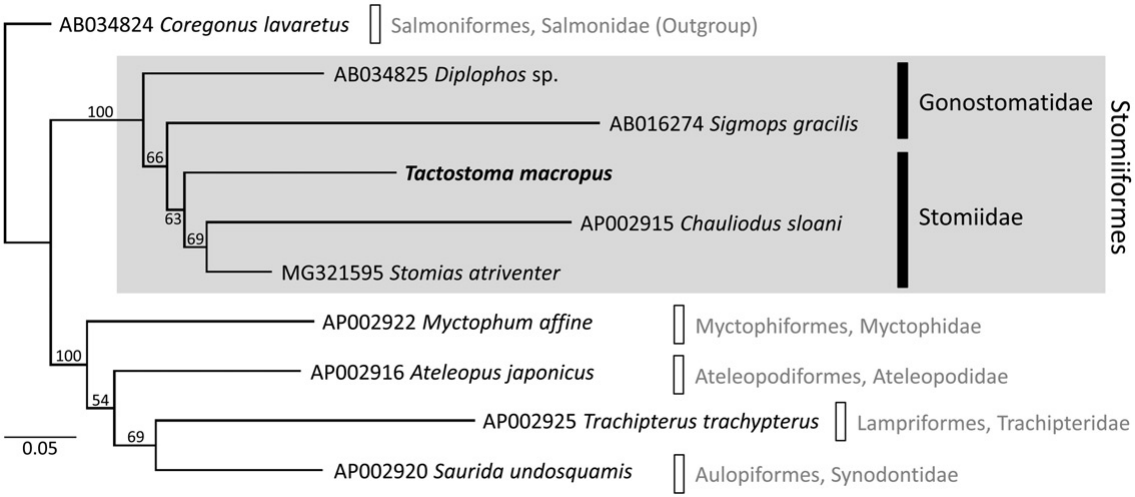
Maximum likelihood (ML) phylogeny of 10 teleost fishes according to the concatenated amino-acid sequences of 13 protein-coding genes (3784 positions). *Tactostoma macropus* is shown in bold. Sequences were aligned separately for each gene using MAFFT version 7.047 (Katoh and Standley 2013) with default parameters. Ambiguously aligned positions were removed using Gblocks Server version 0.91b (Castresana 2000), with all options for less stringent selection. ML analysis was performed in RAxML version 7.2.6 (Stamatakis 2006) using mtREV + G model; nodal support was estimated by 1000 bootstrap replicates. DDBJ/EMBL/GenBank accession numbers are shown for published sequences.
